# Headache in an emergency room

**DOI:** 10.1590/S1516-31802000000300001

**Published:** 2000-05-02

In this issue of the journal, Bigal M, et al^1^ relate their experience of headache in the emergency room of the Hospital of the University of São Paulo at Ribeirão Preto (São Paulo State), a tertiary care facility. They conclude that the ability to solve simple cases of headache at primary care units in this area is very low, and in consequence a great number of cases proceed to the tertiary care facility for medical diagnosis. This finding is at odds with the quality of medical care in this region of the São Paulo State, which has an excellent physician/population ratio and is strongly influenced by the School of Medicine at USP-Ribeirão Preto. This paper, *"Headache in an emergency room in Brazil"* challenges us to discuss how to share the responsibility of primary care units and high-tech hospitals. In this case, the question is: Are headaches a problem for primary care physicians or for neurologists in a hospital?

The first step towards answering the question is to evaluate how common headaches are in Brazil. Few papers have been published on this, although the existing data show a prevalence rate as high as that observed in other countries.^2^ The second step is to evaluate how severe the headache attacks are in Brazil. As the authors pointed out, most patients who sought out the emergency room had migraine, chronic daily headache or tension-type headache as their diagnosis. The final step is to evaluate the cost of treatment at a primary care unit against the approach at an emergency room. In rich countries, e.g. the United States, where the focus of medical care is on high technology, the cost of diagnostic procedures is increasing, but the quality is not keeping pace.

Our conclusion is that most patients with headache must be assisted in a primary care setting. However, we need to discuss why the emergency room is so attractive to most headache sufferers. We may speculate that shifting the complaint to the emergency room is a consequence of a lack of Family Physicians, General Practitioners, General Internal Medicine Specialists, or using the Brazilian expression, " Clínicos Gerais". This kind of physician can diagnoses and treats the majority of headaches within normal clinical practice. This medical doctor would be a senior physician, with great expertise on the main guidelines for treatment of the most common diseases and with specialization in the treatment of minor symptoms. Nowadays, many general practitioners do not have access to these main treatment guidelines. Equipping general practitioners with these guidelines to be used in their routine activities will be a very important decision to make within the field of public health.

In 1988, the International Headache Society (IHS) distributed international criteria for the classification of headache, including the most common symptoms in each type of headache and the laboratory investigation necessary for the diagnosis of primary and secondary headache.^3^ In this manual, the medical diagnosis of primary headache is discussed. It is noted that this is mostly clinical and based on the symptoms that patients complain about. Wide distribution of this manual can diminish the cost and improve the quality of medical care. Only those patients with complex headache or suspected subarachnoid hemorrhage need to be transferred to hospital to permit a broader neurological approach with more specific radiological or laboratory investigation.

Returning to our starting point, a wide-ranging discussion on the most important guidelines in association with a consideration of the minor symptoms can drastically change the primary care diagnosis of some complaints, leaving the more complicated cases to the specialist, when broader investigative support is necessary.
